# Supplementary material for: Identification of photoperiod-induced specific miRNAs in the adrenal glands of Sunite sheep (Ovis aries)
Source: Front Vet Sci. 2022 Jul 22;9:888207. doi: 10.3389/fvets.2022.888207 (PMC9354845; doi:10.3389/fvets.2022.888207)
Supplement: Supplementary file 1 [file Data_Sheet_1.ZIP › Supplementary file/miRDeep2 file.zip!/miRDeep2 file/Novel_Structure/LP42A1/Novel_411.pdf]

Provisional ID	:	NC_019464.2_14801
Score total	:	0.7
Score for star read(s)	:	-1.3
Score for read counts	:	0
Score for mfe	:	0.4
Score for randfold	:	1.6
Score for cons. seed	:	
Total read count	:	55
Mature read count	:	55
Loop read count	:	0
Star read count	:	0

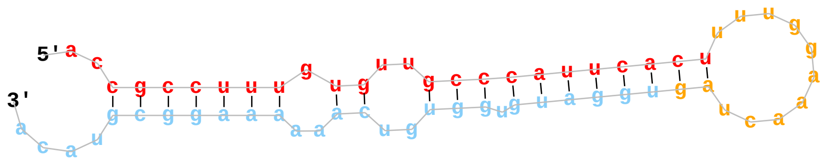

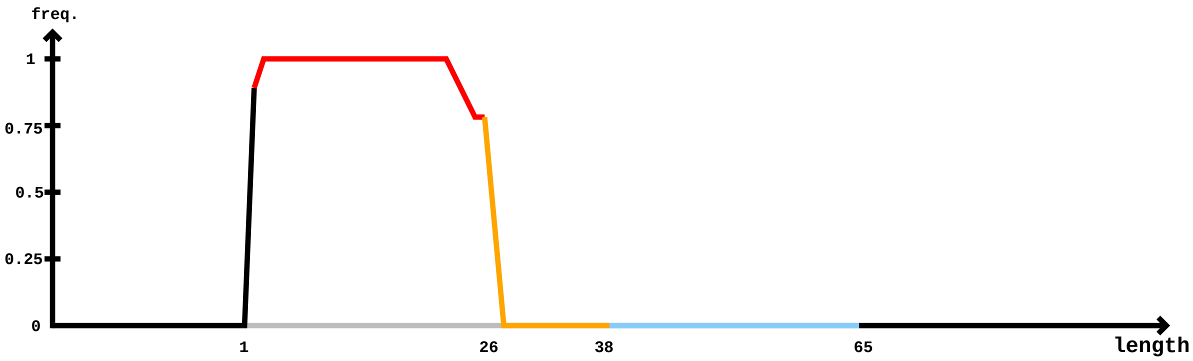

Mature **Star**

```
5'- uuccuuaugcgguuuucuuaccgccuuuguguugcccauucacuuuuggaaacuaguggaugguggugucaaaaaaggcguacacaaaacacuuucgcagcuuuuuuuccugccc   -3'    exp
    ..........(((((.....(((((((.((..((((((((((..........)))))))).)))..))..)))))))).....)))))......((((.........))))..     reads      mm         sample
    ...................accgccuuuguguugcccauu...........................................................................     4          0          seq
    ...................accgccuuuguguugcccauuc..........................................................................     4          0          seq
    ...................accgccuuuguguugcccauuca.........................................................................     4          0          seq
    ...................accgccuuuguguugcccauucacu.......................................................................     32         0          seq
    ...................accgccuuuguguugcccauucacuu......................................................................     5          0          seq
    ....................ccgccuuuguguugcccauucacu.......................................................................     6          0          seq
```
